# Gastric cancer cell growth and epithelial-mesenchymal transition are inhibited by γ-secretase inhibitor DAPT

**DOI:** 10.3892/ol.2014.1980

**Published:** 2014-03-18

**Authors:** LU-CHUN LI, YANG PENG, YAN-MIM LIU, LU-LU WANG, XIAO-LING WU

**Affiliations:** Department of Gastroenterology, The Second Affiliated Hospital of Chongqing Medical University, Chongqing 400010, P.R. China

**Keywords:** epithelial-mesenchymal transition, gastric cancer, Notch1

## Abstract

The Notch signaling pathway may be important in the development and progression of several malignancies. However, the functions of Notch signaling in epithelial-mesenchymal transition (EMT) remain largely unknown. The aim of the present study was to delineate Notch1 expression in gastric cancer (GC) and its function in GC EMT. Using quantitative polymerase chain reaction and western blot analysis, the expression of Notch1 was found to increase in GC cell lines compared with the normal gastric mucosa cell line. In addition, Notch1 expression was found to be downregulated in the non-metastatic-derived GC cell line compared with the metastatic-derived cell line. Furthermore, Notch1 expression was significantly increased in the tumor tissues compared with the adjacent normal mucosa tissues, as well as in patients with metastases than in patients without metastases. To explore the role of the Notch1 signaling pathway in EMT, the GC cell lines, AGS and MKN45, were treated with γ-secretase inhibitor DAPT. Using MTT, Transwell and clonality assays, DAPT was found to inhibit the expression of the Notch1 downstream target, Hes1, and impair the ability of the GC cell lines to migrate, invade and proliferate. The protein levels of the mesenchymal markers, vimentin, neural cadherin and Snail, were decreased; however, the expression of the epithelial marker, epithelial cadherin, was increased in the GC cell lines treated with DAPT. These results indicated that the Notch1 signaling pathway may be important in the development and progression of GC. In conclusion, DAPT inhibits the Notch1 signaling pathway, as well as the growth, invasion, metastasis and EMT of GC cells.

## Introduction

Although there has been an overall worldwide decline in incidence, gastric cancer (GC) remains one of the most common types of cancer and is the second leading cause of cancer-related mortality (1). In China, a large number of GC patients are diagnosed following tumor metastasis and, despite a large number of clinical trials with conventional and targeted therapies, current treatments only offer limited benefits. Thus, strategies are required to overcome this life-threatening disease.

Although a number of molecular markers have been associated with the metastasis of human carcinoma, one of the most important factors contributing to malignancy is the loss of epithelial differentiation. This phenomenon is manifested as an epithelial-mesenchymal transition (EMT), which promotes cancer invasion and metastasis (2). During EMT, the epithelial-specific junction protein, epithelial-cadherin (E-cadherin), is downregulated and mesenchymal proteins, such as neural cadherin (N-cadherin), are upregulated (3). Therefore, epithelial cells become individual, non-polarized, motile and invasive mesenchymal cells (4).

EMT is a dynamic process and is triggered by the interplay of extracellular signals, as well as a number of secreted soluble factors, including transforming growth factor-β, nuclear factor κB, platelet-derived growth factor, Wnt (including a variety of isoforms), microRNAs and others (5–9). Furthermore, the Notch signaling pathway has been reported to be involved in the acquisition of EMT (10).

Notch signaling is known to regulate a number of cellular processes, including cell proliferation, apoptosis, migration, invasion and angiogenesis (11). In addition, Notch expression has been reported to be upregulated in a number of human malignancies (12). However, the function of Notch in the EMT processes of GC remain largely unknown. Therefore, the focus of the present study was to determine the role of the Notch1 signaling pathway in EMT.

## Materials and methods

### Human tissue specimens and cell lines

The human tissue specimens, including 45 samples of human GC (22 samples with metastasis and 23 samples without metastasis) and 25 samples of adjacent normal mucosal tissues, were collected from 70 patients who underwent surgery at the First and Second Affiliated Hospital of Chongqing Medical University (Chongqing, China) between 2011 and 2013. The study complied with the regulations of the Ministry of Health, World Health Organization Research Ethics Review Committee international guidelines for research involving humans and the Declaration of Helsinki on the Ethical Principles for Medical Research Involving Human Subjects. In addition, written informed consent was obtained from the patients prior to the procedures and Institutional Review Board approval was granted from the First and Second Affiliated Hospitals of Chongqing Medical University.

The human GC AGS cell line was obtained from the American Type Culture Collection (Manassas, VA, USA) and the MKN-45 and GES1 cell lines were purchased from the Type Culture Collection of the Chinese Academy of Sciences (Shanghai, China). The cell lines were cultured in RPMI-1640 (Hyclone, Logan, Utah, USA) supplemented with 10% fetal bovine serum (FBS; Hyclone), and maintained at 37°C in a humidified atmosphere of 5% CO_2_/95% air. For the *in vitro* experiment, the cells were treated with γ-secretase inhibitor DAPT (Sigma-Aldrich, St. Louis, MO, USA) at a concentration of 10 μM (13) or with dimethyl sulfoxide (DMSO; as a control) and analyzed after 72 h.

### Immunoblotting

The total protein for the immunoblots was extracted from the cell lines and tissue specimens using the radioimmunoprecipitation assay lysis buffer (Beyotime, Shanghai, China), according to the manufacturer’s instructions. Following the quantification of the protein extracts in a bicinchoninic acid protein assay, equivalent amounts of lysates were resolved using 10% SDS-polyacrylamide gel (Beyotime) electrophoresis and transferred onto a polyvinylidene fluoride membrane (Beyotime). The membrane was then blocked in 5% non-fat milk in Tris-buffered saline (Beyotime) and Tween 20 (Beyotime) for 1 h at 4°C. The blots were then incubated with primary antibodies and subsequently incubated with horseradish peroxidase (HRP)-conjugated secondary antibodies. The signals were then detected by an enhanced chemiluminescence reagent (Millipore, Billerica, MA, USA).

The rabbit monoclonal antibodies against vimentin, E-cadherin and N-cadherin were purchased from Abcam (Cambridge, UK); the mouse monoclonal antibodies against Snail and GAPDH were purchased from BD Biosciences (Franklin Lakes, NJ, USA); and the monoclonal HRP-conjugated goat anti-mouse and anti-rabbit IgG were purchased from Santa Cruz Biotechnology, Inc. (Santa Cruz, CA, USA)

The following antibody dilutions were used: 1:10,000 for anti-Hes1; 1:1,200 for anti-Notch1; 1:1,200 for anti-vimentin; 1:1,200 for anti-E-cadherin; 1:1,200 for anti-N-cadherin; 1:500 for anti-Snail and anti-GAPDH; and 1:7,000 for HRP-conjugated IgG.

### Quantitative polymerase chain reaction (qPCR)

The RNA was purified from cell lines and tissue specimens using RNAiso (Takara Bio, Inc., Shiga, Japan), and cDNA was synthesized using a PrimeScript™ RT reagent kit (Takara Bio, Inc.). The qPCR was performed using the CFX96™ Real-Time PCR Detection system (Bio-Rad, Hercules, CA, USA) with SYBR^®^ Premix Ex Taq™ II (Takara Bio, Inc.). The PCR conditions used were as follows: 95°C for 30 sec followed by 40 cycles of 95°C for 5 sec and 60°C for 30 sec. The results were normalized against β-actin RNA and the sequences of PCR primers used for each of the gene transcripts were as follows: Sense, 5′-TGCCGAACCAATACAACCCTC-3′ and anti-sense, 5′-TGGTAGCTCATCATCTGGGACA-3′ for Notch1; and sense, 5′-CCACGAAACTACCTTCAACTCC-3′ and anti-sense, 5′-GTGATCTCCTTCTGCATCCTGT-3′ for β-actin.

### Cell viability

The cells were seeded into 96-well plates at a density of 5×10^3^ cells/well and incubated overnight. Following the treatment with DAPT at a concentration of 10 μM or with DMSO (as a control), the cells were incubated for 72 h. Next, 20 μl MTT solution (5 mg/ml) was added to the cultures (200 μl) prior to a 4-h incubation at 37°C. Following the removal of the culture medium, the remaining crystals were dissolved in DMSO and the absorbance of the plates were read at 570 nm.

### Clonality assays

For the colony formation assays, the cells treated with DMSO and DAPT were seeded at a low density (1,000 cells/plate) and cultured until visible colonies appeared. The colonies were then stained with Giemsa and counted.

### Migration and invasion assays

AGS and MKN45 (10×10^4^ cells per 500 μl of serum-free media) cells treated with DAPT and DMSO (as a control) were added to the upper chambers, and the lower chambers were filled with 750 μl of media containing 10% FBS. The cells were then incubated for 24 h at 37°C in a humidified atmosphere of 5% CO_2_ in a tissue culture incubator. After 24 h, the non-migrated/invading cells were removed from the upper sides with cotton-tipped swabs. The migrated/invaded cells on the lower sides of the inserts were then stained and the absorbances were read at 560 nm, according to the manufacturer’s instructions.

### Statistical analysis

All experiments were repeated three times and the results were analyzed using SPSS 16.0 software (SPSS, Inc., Chicago, IL, USA). Data are presented as the mean ± standard deviation and group comparisons were performed using Student’s t-test and one-way analysis of variance. P<0.05 was considered to indicate a statistically significant difference.

## Results

### Notch1 expression is upregulated in GC cell lines

**Figure 1:**
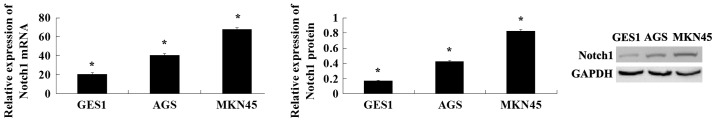
Notch1 expression is upregulated in GC cell lines. Notch1 expression was reduced in GC cell lines (AGS and MKN45) compared with the normal gastric mucosa GES1 cell line. However, Notch1 expression was upregulated in the GC MKN45 cell line derived from metastatic tissue compared with the AGS cell line derived from non-metastatic tissue. Data are presented as the mean ± SD (n=5). ^*^P<0.05, the three groups were compared with each other. GC, gastric cancer.

To explore the expression of Notch1 in human GC cell lines, its expression was analyzed in two cancer cell lines (AGS and MKN45) and in a normal gastric mucosa cell line (GES1). The expression of Notch1 was found to increase in the GC cells compared with the normal gastric mucosa cells. The AGS cells were derived from non-metastatic tissue and the MKN45 cells were derived from metastatic tissue, and Notch1 expression was increased in MKN45 cells compared with the AGS cells (Fig. 1). Thus, Notch1 may be important in GC.

### Notch1 expression is upregulated in GC tissues and increased Notch1 expression is associated with metastatic GC

**Figure 2:**
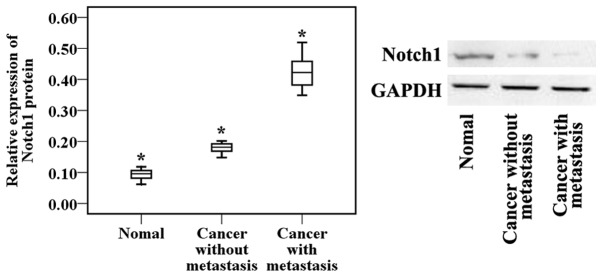
Notch1 expression is upregulated in GC tissues and increased Notch1 expression is associated with metastatic GC. Notch1 expression levels in the adjacent normal mucosa (n=25), non-metastatic GC (n=23) and metastatic GC (n=22) tissues were determined by western blot analysis. Notch1 expression levels were significantly higher in the GC tissues than in the adjacent normal mucosa tissues, as well as in patients with metastasis than in patients without metastasis. Data are presented separately as the mean ± SD for the human samples. ^*^P<0.01, the three groups were compared with each other. GC, gastric cancer.

Therefore, to explore the role of Notch1 in human GC development, its expression levels were detected in 45 human GC tissue samples and 25 adjacent normal mucosa tissue samples. According to the results of the western blot analysis, the expression of Notch1 was significantly upregulated in the tumor tissues compared with the adjacent normal mucosa tissues. Furthermore, to explore whether Notch1 expression is associated with the metastasis of GC, the Notch1 expression levels were examined in 45 gastric tumor samples. These tumors were divided into the following two groups: i) Tumors resected from 22 patients with lymph node or distant organ metastases; and ii) tumors resected from 23 patients without metastases. The western blot analysis also demonstrated that the expression of Notch1 was significantly increased in the patients with metastasis compared with the patients without metastasis (Fig. 2). These results showed that the Notch1 signaling pathway is involved in the development and metastasis of GC.

### γ-secretase inhibitor DAPT prevents the Notch-induced proliferation, migration and invasion of GC cell lines

**Figure 3:**
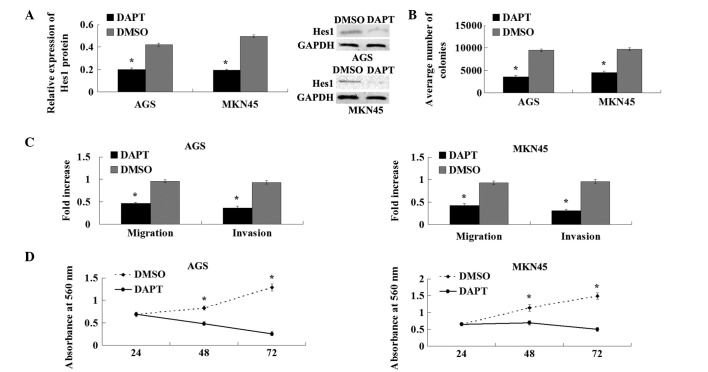
DAPT inhibits gastric cancer cell migration and invasion and downregulates the Notch pathway downstream target, Hes1. (A) AGS and MKN45 cells treated with DAPT and the DMSO control showed a downregulation of the Hes1 protein as determined by western blot analysis. DAPT significantly (B) inhibited the colony-forming abilities, (C) reduced the migration and invasion capacities and (D) inhibited the proliferation abilities of AGS and MKN45 cells compared with the controls. ^*^P<0.05, vs. the control. DMSO, dimethyl sulfoxide.

**Figure 4:**
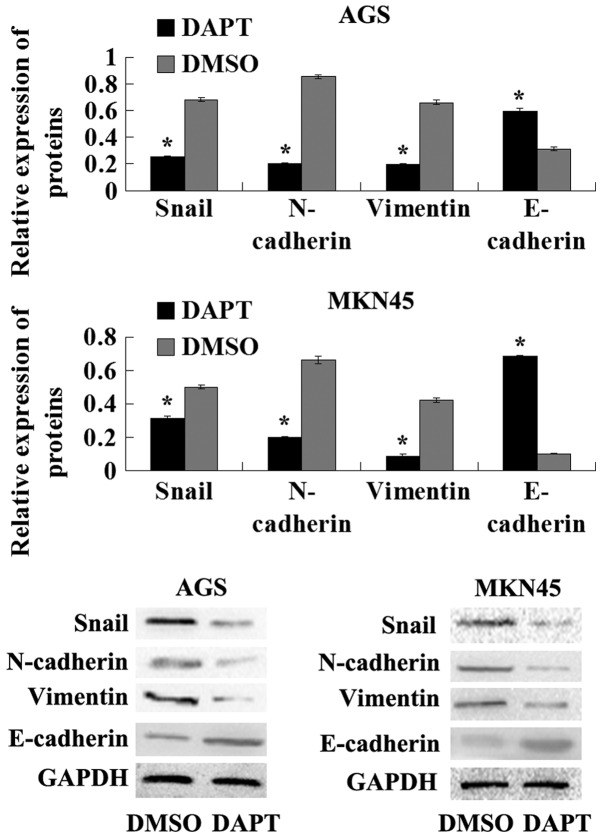
DAPT impairs EMT in AGS and MKN45 cells. EMT markers were analyzed in AGS and MKN45 cells by immunoblotting following treatment with DAPT or the DMSO control. Data are presented as the mean ± standard deviation (n=5). ^*^P<0.05, vs. the controls. DMSO, dimethyl sulfoxide; EMT, epithelial-mesenchymal transition; N-cadherin, neural cadherin; E-cadherin, epithelial cadherin.

To explore the role of the Notch1 signaling pathway in the development and progression of GC, AGS and MKN45 cells were treated with DAPT. The western blot analysis showed that DAPT treatment markedly suppresses the expression of the Notch1 downstream target, Hes1 (Fig. 3). The colony-forming and proliferation abilities in the cells treated with DAPT were also reduced significantly compared with the cells treated with the DMSO control (Fig. 4A and B). The Transwell migration and Matrigel invasion assays demonstrated that DAPT reduces the migration and invasion capacities of AGS and MKN45 cells (Fig. 4C). These results showed that γ-secretase inhibitor DAPT suppresses the Notch1 signaling pathway and inhibits the proliferation, migration and invasion of GC cell lines.

### γ-secretase inhibitor DAPT prevents EMT in GC cell lines

To further explore the molecular mechanism of the inhibition of DAPT on EMT in human GC cell lines, the expression of the epithelial marker, E-cadherin and mesenchymal markers, such as vimentin, N-cadherin and Snail, were examined in AGS and MKN45 cells in the presence of DAPT or DMSO. The results of the western blot analysis showed that the protein levels of N-cadherin, vimentin and Snail were decreased in the cells treated with DAPT. Furthermore, E-cadherin expression was upregulated in the cells treated with DAPT compared with the cells treated with the DMSO control. Overall, these results indicated that the γ-secretase inhibitor DAPT impairs EMT in GC cells.

## Discussion

Emerging evidence suggests that Notch receptors and their ligands are upregulated in cervical, lung, colon, head and neck and renal carcinomas, acute myeloid, Hodgkin’s and large-cell lymphomas, and pancreatic cancer (14–16). Furthermore, the high expression levels of Notch1 and its ligand, Jagged-1, have been associated with a poor prognosis in breast cancer, bladder cancer, leukemia, intrahepatic cholangiocarcinoma and prostate cancer (17–22). In pancreatic cancer cell lines, the activation of Notch1 signaling has been found to contribute to invasion and metastasis by EMT (23,24). In the present study, the expression of Notch1 was found to increase in the GC AGS and MKN45 cell lines compared with the normal gastric mucosa GES1 cell line. In addition, Notch1 expression was significantly higher in the tumor tissues than that in the adjacent normal mucosa tissues, as well as in the metastatic patients compared with the non-metastatic patients. The results showed that Notch1 signaling correlates with the invasion and metastasis of GC and are consistent with the results of several studies that have been previously conducted (25).

The Notch genes encode proteins that are activated by interacting with a family of ligands. Upon activation, Notch is cleaved, releasing the intracellular domain of Notch (ICN) through a cascade of proteolytic cleavages by the metalloprotease tumor necrosis factor-α converting enzyme and the γ-secretase complex (26,27). Therefore, inhibiting the γ-secretase function is likely to prevent the cleavage of the Notch receptor and block the Notch signaling pathway. Furthermore, in pancreatic cancer cell lines, the inhibition of Notch1 signaling prevents migration and invasion (13). In the present study, following treatment of GC cell lines with the γ-secretase inhibitor DAPT, the expression of the Notch1 target gene, Hes1, was significantly decreased, E-cadherin was upregulated and mesenchymal proteins, such as N-cadherin and vimentin, were downregulated. In addition, the inhibition of Notch1 signaling with DAPT significantly decreased the colony formation, migration and invasion of GC cell lines compared with the cells treated with the DMSO control.

A crucial step in impairing GC cell migration and invasion through the inhibition of Notch1 signaling may be the downregulation of E-cadherin expression during the acquisition of the EMT phenotype, which reduces cell-cell adhesion and destabilizes the epithelial architecture. Furthermore, E-cadherin gene repression has been attributed to the function of Snail, which is activated during the acquisition of EMT. Snail may bind to the two E-boxes of the E-cadherin promoter and function as a repressor of E-cadherin expression (28). Therefore, any biological processes that trigger Snail overexpression are likely to downregulate E-cadherin expression, leading to the acquisition of EMT. The effects of Notch1 on E-cadherin expression are mediated through ICN via the regulation of Snail expression. In addition, it has been reported that the overexpression of Notch-1 induces Snail expression, which yields attenuated E-cadherin expression and the acquisition of EMT. Therefore, the γ-secretase inhibitor DAPT can inhibit this process (29). In the present study, the expression of Hes1 was also found to significantly decrease following treatment with the γ-secretase inhibitor DAPT. In addition, Snail expression was downregulated and EMT was impaired in cells treated with DAPT.

In conclusion, the present study identified that the Notch1 signaling pathway is closely associated with the growth, invasion and metastasis of GC. Furthermore, the results demonstrated that the suppression of Notch1 with the γ-secretase inhibitor DAPT restrains the growth, invasion and metastasis of GC by inhibiting EMT.
